# TPX2 Impacts Acetylation of Histone H4 at Lysine 16: Implications for DNA Damage Response

**DOI:** 10.1371/journal.pone.0110994

**Published:** 2014-11-03

**Authors:** Gernot Neumayer, Minh Dang Nguyen

**Affiliations:** Departments of Clinical Neurosciences, Cell Biology & Anatomy, Biochemistry & Molecular Biology, and Hotchkiss Brain Institute, University of Calgary, Cumming School of Medicine, Calgary, Alberta, Canada; Peking University Health Science Center, China

## Abstract

During interphase, the spindle assembly factor TPX2 is compartmentalized in the nucleus where its roles remain largely uncharacterized. Recently, we found that TPX2 regulates the levels of serine 139-phosphoryated H2AX (γ-H2AX) at chromosomal breaks induced by ionizing radiation. Here, we report that TPX2 readily associates with the chromatin in the absence of ionizing radiation. Overexpression of TPX2 alters the DAPI staining pattern of interphase cells and depletion of TPX2 constitutively decreases the levels of histone H4 acetylated at lysine16 (H4K16ac) during G1-phase. Upon ionizing irradiation, this constitutive TPX2 depletion-dependent decrease in H4K16ac levels correlates with increased levels of γ-H2AX. The inversely correlated levels of H4K16ac and γ-H2AX can also be modified by altering the levels of SIRT1, herein identified as a novel protein complex partner of TPX2. Furthermore, we find that TPX2 depletion also interferes with formation of 53BP1 ionizing radiation-induced foci, known to depend on γ-H2AX and the acetylation status of H4K16. In brief, our study is the first indication of a constitutive control of TPX2 on H4K16ac levels, with potential implications for DNA damage response.

## Introduction

The evolutionary conserved targeting protein for *Xenopus* kinesin like protein 2 (TPX2) has been extensively studied as a mitotic factor critical for organization of microtubule, spindle formation, and Aurora A kinase regulation [1]–[9]. During interphase, TPX2 exhibits a stippled distribution pattern with distinct focal enrichments throughout the nucleus [2], [8]. However, TPX2's nuclear functions remain virtually unexplored [8], [10]. Interestingly, *Xenopus laevis* TPX2 co-localizes with condensing chromatin at the transition of interphase to mitosis [4]. A recent report also described a potential heterochromatin protein 1 (HP1) interaction motif in the primary structure of *Arabidopsis thaliana* TPX2 [11]. In addition, ectopic TPX2 forms discrete focal structures that co-localize with interphase chromatin in *Arabidopsis thaliana* [11]. Finally, human TPX2 is found in complex with BRCA1 [12], [13], a modifier of chromatin architecture [14]. Taken together, these studies suggest that TPX2 may be implicated in chromatin biology.

We have recently uncovered a novel function for TPX2 in DNA damage response [15]. DNA damage response activates so-called ‘checkpoint pathways’ that halt the cell cycle in order to repair the insulted DNA. Alternatively, DNA damage response induces cell death or senescence if the damage is too severe [16]–[20]. Together, this ensures genomic stability whereas inability to mount a proper response to DNA damage can promote development of cancer [21], [22]. A central step in the cellular response to DNA double strand breaks is the formation of γ-H2AX, i.e. phosphorylation of histone H2AX (on serine139 in human) in chromatin adjacent to DNA ruptures [16], [23]–[25]. Our previous study demonstrated that the levels of TPX2 inversely correlate with the levels of γ-H2AX generated by the ATM kinase [15]. Particularly during G1-phase of the cell cycle, a dramatic increase in H2AX phosphorylation was observed in the absence of TPX2 upon treatment with clastogenic ionizing radiation [15]. However, the number of ionizing radiation-induced DNA double strand breaks was unperturbed in TPX2-depleted cells [15]. Instead, these cells exhibited an increased intensity of γ-H2AX ionizing radiation-induced foci (i.e. the microscopic visualization of γ-H2AX at DNA breaks), indicating hyper-phosphorylation of H2AX at DNA lesions [15]. TPX2's active function in DNA damage response is further highlighted by its accumulation on chromatin that surrounds chromosomal breaks [15].

It remains unknown how TPX2 impacts the levels of γ-H2AX. While numerous factors directly controlling H2AX phosphorylation (i.e. kinases and phosphatases [25]–[32]) have been identified, other parameters, e.g. the type of chromatin [33], [34], also influence γ-H2AX formation. The architecture of the chromatin is, in part, established by the counteracting actions of histone acetyl transferases (HAT) and histone deacetylases (HDAC) [34]–[36]. Importantly, both types of enzymes have been implicated in DNA damage response [37]–[42]. For example, the HATs MOF and TIP60 induce acetylation (ac) of histone H4 on lysine16 (H4K16) during DNA damage response and are important for DNA repair and cellular survival [37], [42]–[44]. On the other hand, the SIRT1 HDAC antagonizes these actions by deacetylating H4K16ac and promoting the proteasomal degradation of MOF [40], [45], [46]. Although a transient delay (up to 15 min upon genomic insult) in H2AX phosphorylation was observed in cells with decreased H4K16ac levels [43], it remains unknown whether this histone modification further impacts γ-H2AX signaling. Downstream of H2AX phosphorylation and dependent on the acetylation status of H4K16, 53BP1 accumulates on chromatin flanking DNA breaks. This matures the DNA damage response and promotes DNA repair and cellular survival [16], [42], [47].

In the present study, we report that overexpression of TPX2 alters the DAPI staining pattern of interphase cells. Furthermore, depletion of TPX2 decreases H4K16ac levels. These phenotypes are observed in the absence of exogenously induced DNA damage. Upon ionizing irradiation, the constitutive TPX2 depletion-dependent decrease in H4K16ac levels correlates with increased levels of γ-H2AX. Altering the levels of SIRT1, herein identified as a novel protein complex partner of TPX2, also changes both H4K16ac and γ-H2AX levels in an inverse manner. Together, these data indicate a balance between H4K16ac and γ-H2AX levels that can be modified by TPX2 and SIRT1. Finally, TPX2 depletion also interferes with 53BP1 ionizing radiation-induced foci formation, an event that depends on H4K16ac and γ-H2AX levels. Our study indicates that TPX2 influences the chromatin environment with potential implications for DNA damage response.

## Material and Methods

### Cell cultures, transfection, and induction of TPX2 miRNA

HeLa (ATCC), HeLa EM2-11-TPX2 (a kind gift from Dr. Oliver J. Gruss-University of Heidelberg; [48]), and MCF7 (ATCC) cells were maintained in DMEM containing 10% fetal bovine serum. Transfection with plasmid DNA or oligonucleotides was done using Lipofectamine 2000 Reagent (Invitrogen) or HiPerFect Transfection Reagent (Qiagen). TPX2 miRNA expression in HeLa EM2-11-TPX2 cells was induced by addition of 1 µg doxycycline/ml of medium [15].

### Cell cycle synchronization and flow cytometry

HeLa EM2-11-TPX2 cells were plated 24 h before being subjected to the following synchronization procedure (i.e. double thymidine block): Treatment with thymidine (2 mM) for 20 h was followed by a passage into thymidine-free media for 15 h and subsequent treatment with thymidine (2 mM) for an additional 14 h. To induce expression of the TPX2 targeting miRNA, doxycycline was added to the media in parallel with the second thymidine treatment as indicated. After the double thymidine block, cells were released into thymidine-free media to allow synchronous cell cycle progression. Cells were then treated with 10 Gy of ionizing radiation (or left untreated) 11 h, 12 h, and 13 h after release from the double thymidine block (i.e. while progressing through G1-phase). Subsequently, cells were incubated for 1 h of recovery, harvested by trypsinization, and analyzed by Western blot. An aliquot (20%) of non-irradiated control cells was fixed with ethanol, stained with propidium iodide, and used for flow cytometry-based cell cycle profiling to ensure equal cell cycle synchronicity in all individual sample populations.

### Chromatin fractionation

Cells were rinsed twice with PBS (37°C) and harvested in ice cold NETN buffer [150 mM NaCl, 1 mM EDTA, 50 mM Tris-Cl pH 7.4, 1% NP40, and 1x protease inhibitor cocktail complete Mini-EDTA free (Roche)]. The obtained cell extracts were sonicated once for 5 seconds with a Sonic Dismembrator Model 100 at Level 4. The insoluble chromatin fraction was pelleted for 20 minutes at 4°C at maximum speed in a table top centrifuge. The soluble NETN fraction was analyzed and nuclear lamins but no histones were found to be contained in this NETN fraction. The insoluble chromatin fraction (containing the histones but no nuclear lamins) was washed twice in 1 ml NETN buffer. Solubilization of the chromatin fraction was achieved by addition of 1% SDS in PBS followed by one freeze and thaw cycle at −80°C, incubation at 95°C for 15 minutes, and sonication for 15 seconds. Protein concentrations were measured with the Bio-Rad DC protein assay (chromatin fraction) or Bio-Rad Protein (Bradford) assay (NETN fraction).

### Co-immunoprecipitations

Co-immunoprecipitation experiments have been described previously [15] and were carried out in the absence or presence of 50 µg/ml ethidium bromide (EtBr). Antibodies specific for TPX2 (a kind gift from Dr. Oliver J. Gruss-University of Heidelberg; [2]) or SIRT1 (Upstate) were used.

### Generation of constructs and RNAi sequences

His-TPX2 and GFP-TPX2 constructs have been generated previously [15]. The Flag-SIRT1 encoding construct was a kind gift of Dr. David Sinclair from Harvard Medical School. Targets of used RNAi sequences are as follows: TPX2 siRNA (5′-AAGAAUGGAACUGGAGGGCUU-3′), TPX2 miRNA (5′-CCGAGCCUAUUGGCUUUGAUU-3′), and SIRT1 siRNA (5′-AAGAUGAAGUUGACCUCCUCA-3′) [2], [48], [49]. A random siRNA sequence without homology to any known mRNA or no induction of the TPX2 miRNA were used as control conditions.

### Immunofluorescence staining and microscopy analysis

Cells were fixed (paraformaldehyde), permeabilized (Triton X-100), blocked (BSA), and stained with antibodies against H4K16ac (Abcam), 53BP1 (Novus Biologicals), and the Xpress-tag encoded by His-TPX2 as per standard laboratory procedures. Nuclei were counterstained with DAPI. Images were acquired with a Nikon Eclipse TE2000-E confocal microscope.

### Ionizing radiation

Treatment with ionizing radiation was performed using a source of Cs^137^ from a MDS Nordion Gammacell 1000.

### Western blotting

Protein concentration was determined by the Bradford procedure or the Bio-Rad DC assay (Bio-Rad Laboratories, Hercules, CA). Proteins were separated on SDS-PAGE and blotted on a PVDF membrane for western blot analysis. Membranes were developed with antibodies specific for 53BP1 (Novus Biologicals), Actin (Chemicon), GAPDH (Abcam), H2AX (Abcam), γ-H2AX (Millipore), H4/H4K16ac (Abcam), H3K9ac (Upstate), H3K56ac (Upstate), H3 (Abcam), Lamin B (Calbiochem), SIRT1 (Upstate), and TPX2 (184, Novus Biologicals). Signals from western blots were quantified with the Quantity-One software from Bio-Rad. Signals were normalized with levels of the non-phosphorylated/acetylated form of the protein of interest.

## Results

### TPX2 associates with the chromatin and TPX2 overexpression alters the DAPI staining pattern

**Figure 1:**
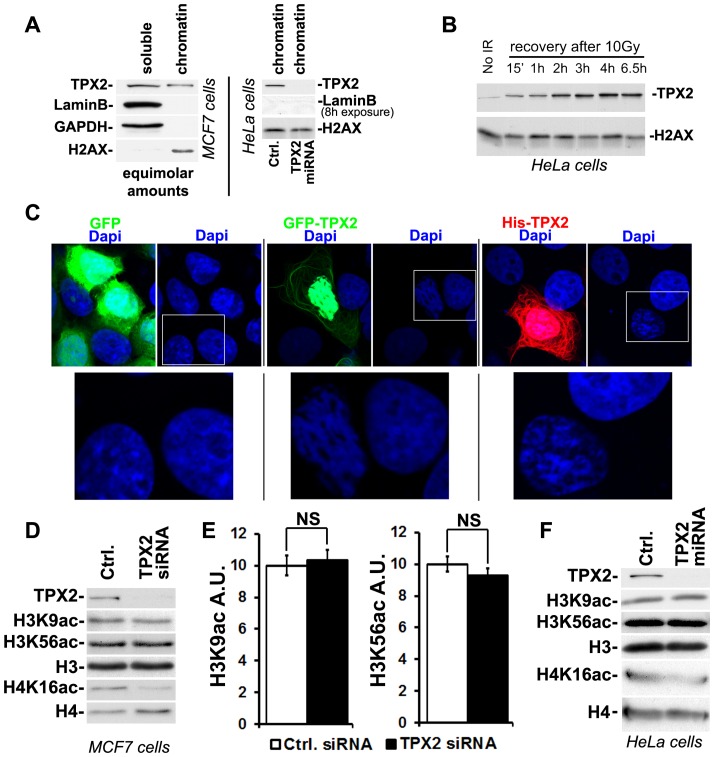
TPX2 is constitutively associated with chromatin and impacts the DAPI staining pattern and H4K16ac levels. (A) Although the majority of TPX2 is found in the soluble fraction (see Material and Methods), a small but clearly detectable sub-population of TPX2 constitutively associates with stringent chromatin fractions obtained from MCF7 cells (left panel) or HeLa cells (right panel). These chromatin fractions contain histones but not nuclear LaminB. Upon expression of an inducible TPX2 targeting miRNA (or upon transfection with siRNA; see D) the protein was depleted from chromatin fractions. Ctrl: control cells with no induction of TPX2 miRNA. (B) TPX2 gets enriched in chromatin fractions isolated from HeLa cells after treatment with 10 Gy of ionizing radiation. Note the constitutive association of TPX2 with the chromatin in non-irradiated cells. Levels of H2AX were used as a loading control. (C) Overexpression of GFP-TPX2 or His-TPX2 causes abnormal DAPI staining in MCF7 cells compared to surrounding non-transfected cells or cells transfected with GFP. This is indicative of changes in chromatin structure. Enlargements of white frames are shown. In agreement with previous reports, overexpressed TPX2 is mostly found in the nucleus but also associates with the cytoskeleton [2]. (D-F) Depletion of TPX2 by siRNA (D) or miRNA (F) causes a decrease in H4K16ac levels whereas the levels of H3K9ac and H3K56ac remain unchanged. (E) Quantification of H3K9ac and H3K56ac levels from MCF7 cells transfected with control or TPX2 siRNA (n = 4 independent experiments each; p(*t* test)>0.05; NS: non significant; Error bars represent SE). Stripping of western blots and re-development with antibodies specific for H3 and H4 ensured equal loading. See text for details.

Since TPX2 partially co-localizes with DNA during interphase [2], [15], we investigated a potential constitutive association of TPX2 with the chromatin. In the absence of exogenously induced DNA damage, TPX2 is readily found in chromatin fractions obtained from MCF7 cells and HeLa cells (Fig.1A). These fractions contain histone proteins but no nuclear lamins (Fig. 1A), indicating high stringency of the chromatin purification method. Expression of a doxycycline-inducible TPX2 targeting miRNA in HeLa cells [48] or transient transfection of MCF7 cells with a TPX2 targeting siRNA depleted the protein from these chromatin fractions (Figs.1A, 1D, 1F). Efficiencies and specificities of the two TPX2 targeting RNAi sequences have been determined previously [15], [48]. The use of two independent RNAi approaches in two different cell lines provides strong evidence that the observed protein is indeed endogenous TPX2. It is noteworthy that the abundance of TPX2 in chromatin fractions increases after treatment with ionizing radiation (Fig.1B). This finding is in agreement with our published work documenting the recruitment of TPX2 to DNA double strand breaks [15].

Compatible with the presence of TPX2 in chromatin fractions, we found that overexpression of either His-TPX2 or GFP-TPX2 in non-irradiated MCF7 cells causes abnormal DAPI (4′,6-diamidino-2-phenylindole) staining patterns (Fig.1C). In these cells, the DAPI staining is more structured and compartmentalized than the uniformly distributed DAPI signal found in surrounding non-transfected control cells or cells expressing GFP (Fig.1C).

### TPX2 depletion decreases the levels of H4K16ac

**Figure 2:**
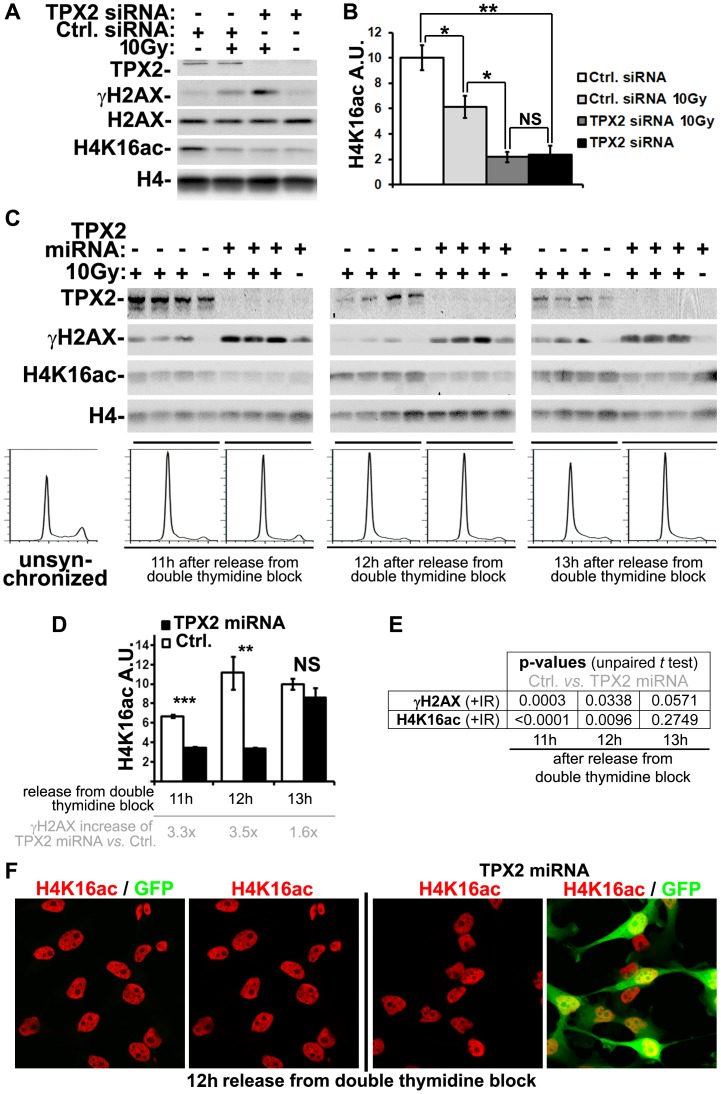
TPX2 selectively regulates the levels of H4K16ac during G1-phase. (A) Depletion of TPX2 by siRNA in MCF7 cells causes a constitutive decrease in H4K16ac levels that correlates with the known increase in ionizing radiation-dependent (10 Gy) γ-H2AX levels [15]. Levels of H2AX and H4 were used as loading controls. (B) Quantification of H4K16ac levels from control (Ctrl) and TPX2 siRNA transfected MCF7 cells with and without ionizing radiation treatment (10 Gy). Note that H4K16ac levels decrease after treatment with ionizing radiation in control siRNA transfected cells whereas TPX2-depleted cells have a constitutive decrease in H4K16ac levels. See text for details (n = 3 independent experiments; Error bars represent SE). (C) HeLa cell cultures enriched for G1-phase cells via release from a double thymidine block exhibit constitutively decreased levels of H4K16ac and increased ionizing radiation-dependent levels of γ-H2AX upon depletion of TPX2 compared to controls (no TPX2 miRNA induction). Flow cytometry based cell cycle profiles (bottom histograms) derived from the non-irradiated cell cultures analyzed by western blots are shown. Note that the TPX2 depletion-dependent γ-H2AX and H4K16ac phenotypes are particularly pronounced 11 h and 12 h after release. During G1/S transition (i.e. 13 h after release), γ-H2AX and H4K16ac levels start to normalize in TPX2-depleted cells. See text for details. (D) Quantification of H4K16ac levels after irradiation throughout G1-phase from control (Ctrl) and TPX2 miRNA expressing HeLa cells (n = 3 independent experiments; Error bars represent SE). The relative increase of γ-H2AX levels upon TPX2 depletion compared to controls is shown for each time point (light grey). (E) p-values (unpaired student's *t* test) describing differences in γ-H2AX levels (n = 3 independent experiments) or H4K16ac levels (n = 3 independent experiments), respectively, between control (Ctrl) and TPX2 miRNA expressing HeLa cells at indicated time points after release from a double thymidine block. Note that the statistically significant (i.e. p<0.05) increase in γ-H2AX levels and decrease in H4K16ac levels upon TPX2 depletion is attenuated at the G1/S transition (i.e. 13 h after release). Levels of H4 and H2AX were used to normalize for loading. NS: non significant, * p<0.05, ** p<0.01, *** p<0.001; IR: ionizing radiation. (F) No apparent global decrease in acetylated H4K16 in TPX2-depleted cells 12 h after release from a double thymidine block (i.e. G1-phase). Expression of the inducible TPX2 miRNA also triggers expression of a GFP reporter.

We previously reported that TPX2 regulates phosphorylation of H2AX upon ionizing irradiation [15]. In addition to H2AX, several other histones are also post-translationally modified during DNA damage response. Notably, the acetylation status of H3K9, H3K56, and H4K16 is changed upon breakage of chromosomes [37], [42], [47], [50]. In light of results showing that ectopic expression of TPX2 alters DAPI staining patterns in non-irradiated cells (Fig.1C), we determined whether TPX2 also affects post-translational modification of histones in the absence of exogenously induced DNA damage. As previously shown, no substantial induction of γ-H2AX was observed in TPX2-depleted cells before treatment with ionizing radiation ([15] and Fig.2A). In non-irradiated MCF7 cells, the levels of H3K9ac and H3K56ac remained unchanged upon TPX2 depletion by siRNA (Fig.1D-E). Intriguingly, the levels of H4K16ac markedly decreased in these cells (Δ∼76%; p(*t* test) = 0.003; 3 independent experiments; Fig.1D and Fig.2A-B for quantifications). To ensure specificity of this phenotype, we also examined H3K9ac, H3K56ac, and H4K16ac levels in HeLa cells depleted of TPX2 by miRNA. Consistently, we observed a substantial decrease in H4K16ac levels in these cells whereas H3K9ac and H3K56ac levels remained unchanged (Fig.1F). Thus, TPX2 impacts the levels of H4K16ac independently of DNA damage in two different cell types.

### The TPX2 depletion-dependent decrease in H4K16ac is unaffected by ionizing irradiation but correlates with increased γ-H2AX during DNA damage response

Since acetylation of H4K16 is modulated upon genomic insult [37], [47], we next sought to determine whether the constitutive TPX2 depletion-dependent decrease in H4K16ac levels (Fig.1) is affected by ionizing irradiation. In agreement with recent findings [47], we found that H4K16ac levels in control MCF7 cells were slightly decreased after treatment with 10 Gy of ionizing radiation (Fig.2A). This phenotype was consistent and statistically significant [Fig.2B; control siRNA - IR (10.0+/−1.0) vs. control siRNA + IR (6.1+/−0.9); p(*t* test) = 0.044; group (mean of H4K16ac +/−SE, A.U.); n = 3 independent experiments; IR: ionizing radiation]. However, non-irradiated MCF7 (and HeLa; Fig.2C) cells depleted of TPX2 by siRNA (or miRNA; Fig.2C) already exhibited significantly lower H4K16ac levels than non-irradiated or irradiated control cells [Fig.2A-B; control siRNA - IR (10.0+/−1.0) vs. TPX2 siRNA - IR (2.4+/−0.7); p(*t* test) = 0.003; group (mean of H4K16ac +/−SE, A.U.); n = 3 independent experiments]. Upon treatment with ionizing radiation, TPX2-depleted cells did not exhibit further decrease in H4K16ac levels [Fig.2A-B; TPX2 siRNA - IR (2.4+/−0.7) vs. TPX2 siRNA + IR (2.2+/−0.4); p(*t* test) = 0.831; group (mean of H4K16ac +/−SE, A.U.); n = 3 independent experiments]. We conceive that in the absence of exogenously caused genomic insult, TPX2 depletion readily decreases H4K16ac to levels that are not further reduced by ionizing irradiation (see Discussion).

Intriguingly, we found that the TPX2 depletion-triggered decrease in H4K16ac levels correlates with an increase in γ-H2AX levels after treatment with ionizing radiation (Fig.2A). Because TPX2's DNA damage response function is particularly evident during G1-phase (see [15]), we next determined the TPX2-dependent levels of H4K16ac at this cell cycle stage. To do so, we utilized the HeLa cell line expressing a doxycycline-inducible TPX2 miRNA and synchronized these cells with a double thymidine block [15], [48]. Long-term depletion of TPX2 is known to impact cell cycle progression [2], [8]. Therefore, we chose a minimal TPX2 knockdown time of less than 27h for these experiments. Our previously published data indicates that HeLa cell cultures are synchronized for S-phase, G2-phase, and M-phase at 2 h, 6 h, and 9 h after release from a double thymidine block. G1-phase occurs from 11 h-12 h after release [15]. In this study, we found that irradiated TPX2-depleted G1-phase-enriched cell cultures with 3.3–3.5 fold elevated levels of γ-H2AX exhibit significantly decreased levels of H4K16ac compared to control cells [Fig.2C-D; 11 h after release: control + IR (66.7+/−1.6) vs. TPX2 miRNA + IR (34.7+/−0.9); 12 h after release: control + IR (111.4+/−16.6) vs. TPX2 miRNA + IR (33.7+/−1.5); group (mean of H4K16ac +/−SE, A.U.); n = 3 independent experiments]. Flow cytometry-based cell cycle profiling ensured that control and TPX2 miRNA expressing cultures exhibit similar cell cycle profiles with similar enrichment of G1-phase cells 11 h (control: 82.6%; TPX2 miRNA: 78.3% G1-phase cells) and 12 h (control: 81.9%; TPX2 miRNA: 81.0% G1-phase cells) after release. In line with results from unsynchronized MCF7 cell cultures (Fig.2A-B), the TPX2 depletion-dependent decrease in H4K16ac levels observed in G1-phase HeLa cells appears to be independent of ionizing irradiation (Fig.2C).

Of note, 13 h after release from the double thymidine block the percentage of cells in G1-phase decreased (control: 71.4%; TPX2 miRNA: 76.4% G1-phase cells) and cells started to enter S-phase (control: 27.2%; TPX2 miRNA: 21.2% S-phase cells). 15 h after release ∼40% of cells had entered S-phase, indicating the completion of one synchronous cell cycle (data not shown). Parallel with the transition into S-phase at 13 h after release from the double thymidine block, the decrease in H4K16ac levels in TPX2-depleted HeLa cells became attenuated [Fig.2C-D; control + IR (100.0+/−5.5) vs. TPX2 miRNA + IR (85.9+/−9.7); group (mean of H4K16ac+/−SE, A.U.); n = 3 independent experiments]. This attenuation of the H4K16ac phenotype was accompanied by a diminished magnitude and reduced statistical significance of the TPX2 depletion-dependent γ-H2AX increase (Fig.2C-E). The latter is in agreement with our previously published data documenting that TPX2 depletion has no effect on γ-H2AX in cell cycle phases other than G1 and G0 [15].

In brief, our results indicate that TPX2 constitutively affects the levels of H4K16ac in G1-phase. During DNA damage response, the levels of H4K16ac and γ-H2AX exhibit an inverse correlation. Therefore, the ionizing radiation-independent impact of TPX2 on H4K16ac levels (Figs.1D, 1F, 2A-C) may affect the phosphorylation of H2AX once DNA damage response is launched. Intriguingly, single cell analysis via confocal microscopy did not reveal a notable decrease in global acetylation of H4K16 upon TPX2 depletion (Fig.2F). This suggests that TPX2-dependent changes in H4K16ac levels may be restricted to certain genomic loci. Further studies are necessary to decipher where exactly in the genome TPX2 impacts the levels of H4K16ac (see Discussion).

### SIRT1 impacts the levels of H4K16ac and γ-H2AX and associates with TPX2

**Figure 3:**
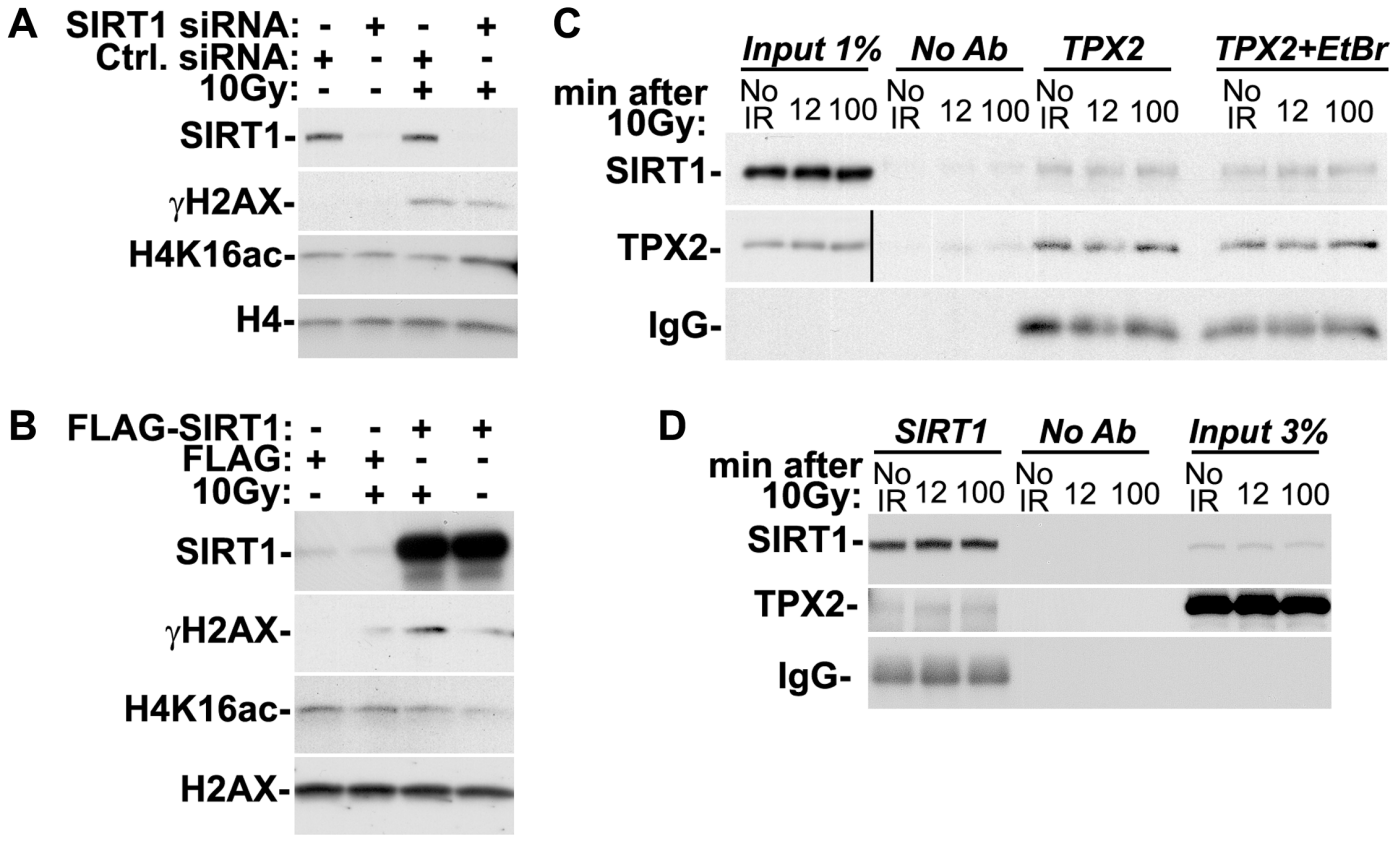
SIRT1 modulates the levels of H4K16ac and γ-H2AX and is in complex with TPX2. (A) siRNA-mediated loss of SIRT1 in HeLa cells increases H4K16ac levels and decreases ionizing radiation-dependent γ-H2AX levels when compared to controls. (B) Overexpression of SIRT1 in MCF7 cells decreases H4K16ac levels and increases ionizing radiation-dependent γ-H2AX levels when compared to controls. (C) Co-immunoprecipitations with TPX2 antibodies from HeLa cells with and without ionizing radiation treatment as indicated and in the absence or presence of ethidium bromide (EtBr). (The Input for TPX2 is from a longer exposure of the same blot.) (D) Co-immunoprecipitations with SIRT1 antibodies from HeLa cells with and without ionizing radiation treatment as indicated. Beads without antibodies (No Ab) were used as controls. Cells were treated with 10 Gy (or left untreated) and harvested after 1h recovery (A-B) or as indicated (C-D). See text for details. Levels of H2AX and H4 were used as loading controls. IR: ionizing radiation.

H4K16ac is a substrate of SIRT1 HDAC [40], [45], [46]. Interestingly, *SIRT1* knockout mice display increased levels of H4K16ac that correlate with decreased levels of ionizing radiation-triggered γ-H2AX [36]. The increase in H4K16ac levels in these animals is presumably due to loss of SIRT1 HDAC activity [36], [46]. In agreement with these data we observed that siRNA-mediated depletion of SIRT1 in HeLa cells increases H4K16ac levels and decreases γ-H2AX levels compared to controls (Fig.3A). Conversely, overexpression of SIRT1 in MCF7 cells results in decreased H4K16ac levels that correlate with increased levels of ionizing radiation-triggered γ-H2AX compared to controls (Fig.3B). Note that these MCF7 cells are caspase-3-deficient and do not undergo ionizing radiation-induced apoptosis [51], [52]. The observed increase in γ-H2AX levels upon SIRT1 overexpression is therefore not an epiphenomenon of apoptosis, known to also induce γ-H2AX during apoptotic DNA fragmentation [53]. Thus, our data reveal an inverse correlation between the levels of H4K16ac and γ-H2AX. Both TPX2 and SIRT1 can modulate the levels of these post-translationally modified histones (Figs. 2A-E and 3A-B).

In the absence of reports or sequence motifs suggesting an enzymatic activity intrinsic to TPX2, we hypothesized that TPX2 may be part of a regulatory complex that controls the levels of H4K16ac and γ-H2AX. SIRT1 may be a member of this complex since it also modifies H4K16ac and γ-H2AX levels (Fig.3A-B). In support of this hypothesis we found that TPX2 antibodies co-immunoprecipitated a subpopulation of SIRT1 (Fig.3C). Furthermore, SIRT1 antibodies also co-immunoprecipitated a subpopulation of TPX2 (Fig.3D). The yield of SIRT1 in the TPX2 co-immunoprecipitations was not affected by the presence of ethidium bromide, suggesting that the association between TPX2 and SIRT1 is not mediated by chromatin (Fig.3C). Finally, ionizing irradiation did not impact the association between TPX2 and SIRT1 in these co-immunoprecipitation experiments (Fig.3C-D). The significance of the TPX2/SIRT1 interaction is analyzed in the discussion.

### TPX2 depletion causes defects in 53BP1 recruitment to exogenously induced chromosomal breaks

The recruitment of 53BP1 to DNA double strand breaks occurs downstream of γ-H2AX signaling and is dependent on the acetylation status of H4K16 [16], [20], [42], [47], [54], [55]. Since TPX2-depleted cells exhibit altered levels of γ-H2AX [15] and H4K16ac (Fig.1–2), we hypothesized that 53BP1 ionizing radiation-induced foci formation is also disturbed in these cells.

**Figure 4:**
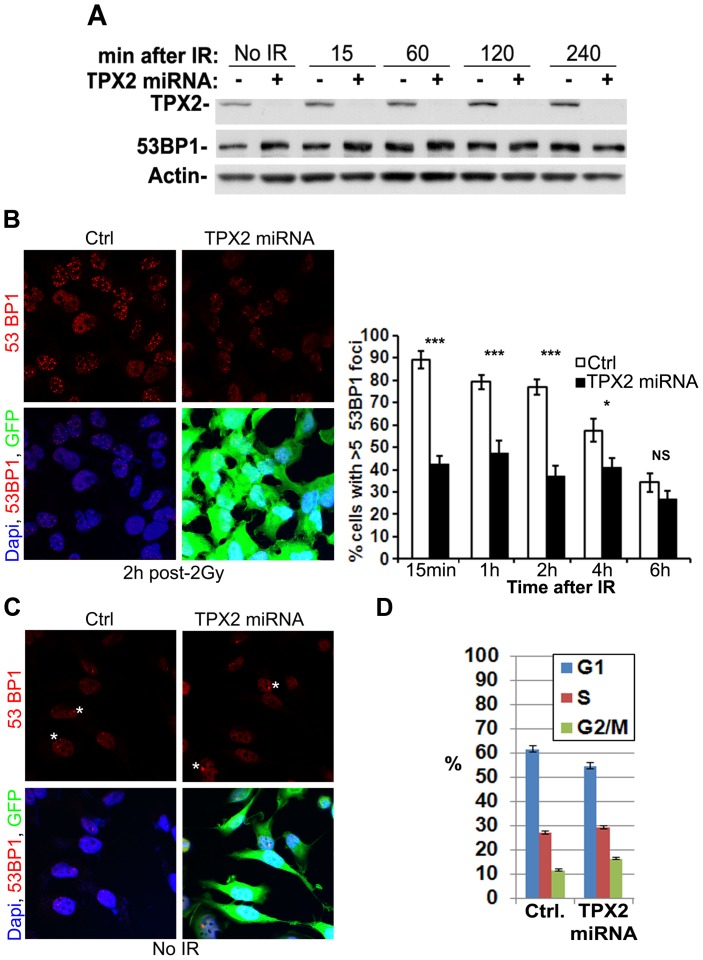
Depletion of TPX2 causes defects in 53BP1 ionizing radiation-induced foci formation. (A) The protein level of 53BP1 is not affected by miRNA-mediated depletion of TPX2. Levels of actin were used as loading controls. (B) Doxycycline-induced expression of TPX2 miRNA significantly decreases the percentage of HeLa cells with more than five 53BP1 ionizing radiation-induced foci 15 min to 2 h after 2 Gy when compared to non-induced controls (Ctrl). Representative images of cells with 53BP1 ionizing radiation-induced foci 2 h after irradiation are shown (left). Doxycycline also induces expression of GFP reporter. Three independent experiments were performed and 18–20 pictures with an average of 16 cells per picture were analyzed per condition for each time point. Data are compiled in bar chart (right): [15 min: control (89.1+/−4.0) vs. TPX2 miRNA (42.6+/−3.6), p<0.001, n = 18; 1 h: control (79.1+/−3.3) vs. TPX2 miRNA (47.6+/−5.3), p<0.001, n = 20; 2 h: control (76.9+/−3.6) vs. TPX2 miRNA (37.2+/−4.5), p<0.001, n = 20; 4 h: control (57.5+/−5.0) vs. TPX2 miRNA (41.0+/−4.2), p<0.05, n = 20; 6 h: control (34.2+/−4.3) vs. TPX2 miRNA (26.7+/−3.9), p>0.05, n = 18; group (mean % of cells with more than five 53BP1 ionizing radiation-induced foci +/−SE); unpaired *t* test]. See text for details. (C) 53BP1 accumulates at infrequent endogenous chromosomal breaks (indicated by asterisks; no ionizing radiation treatment) in presence or absence of TPX2. (D) Cell cycle profiles of control and TPX2 miRNA expressing HeLa cell cultures obtained via flow cytometry (n = 2). Note that the slight ∼5% increase in the G2/M fraction upon TPX2 depletion can not account for the defect in 53BP1 ionizing radiation-induced foci formation exhibited by ∼55% of TPX2 miRNA expressing cells. See text for details. Error bars represent SE in (B) and SDEV in (D). n =  # of independent experiments; NS: non-significant, * p<0.05, ** p<0.01, *** p<0.001; IR: ionizing radiation.

We first analyzed the total amount of 53BP1 in TPX2-depleted cells and found no change in 53BP1 protein levels before and after treatment with ionizing radiation compared to controls (Fig.4A). We then performed a time-course analysis of 53BP1 ionizing radiation-induced foci formation in HeLa cells expressing the doxycycline-induced TPX2 targeting miRNA [48]. 53BP1 ionizing radiation-induced foci formation was significantly impaired in TPX2-depleted cells from 15 min to 2h after an irradiation dose of 2 Gy (Fig.4B). However, 53BP1 focus formation is not inhibited *per se* in the absence of TPX2. Infrequent endogenous DNA double strand breaks that arise at so called “fragile sites” in the absence of ionizing radiation treatment [56]-[58] still recruit 53BP1 upon TPX2 miRNA expression (Fig.4C).

Since depletion of TPX2 has been associated with mitotic arrest in HeLa cells [2], we determined whether the TPX2 depletion-dependent 53BP1 phenotype is the result of increased numbers of mitotic cells, known to exclude 53BP1 from their repair foci [59], [60]. Although we found a modest ∼5% increase of the G2/M population upon TPX2 depletion via flow cytometry-based cell cycle profiling (Fig.4D), this slight increase can not account for the defect in 53BP1 ionizing radiation-induced foci formation exhibited by more than 55% of TPX2-depleted cells (Fig.4B).

## Discussion

The nuclear functions of TPX2 are poorly understood. In the present study, we found that TPX2 associates constitutively with the chromatin and that TPX2 overexpression alters the DAPI staining pattern (Fig.1). Importantly, depletion of TPX2 decreases selectively the levels of H4K16ac (Figs.1–2). This phenotype is particularly evident in cell cultures synchronized at G1-phase (Fig.2) and thus, it is not an artifact of changed cell cycle profiles. Taken together, our results indicate a role for TPX2 in chromatin biology.

Although the significance of TPX2-dependent H4K16ac levels in unperturbed cell cycles remains to be investigated, we propose that this histone modification may impact DNA damage response. Specifically, in TPX2-depleted G1-phase cells the constitutive decrease in H4K16ac levels is paralleled by an ionizing radiation-dependent increase in γ-H2AX levels. These correlating TPX2-dependent H4K16ac and γ-H2AX phenotypes are simultaneously attenuated at the end of G1-phase (Fig.2). Additional evidence for a link between H4K16ac and γ-H2AX levels is provided by our observation that SIRT1 can impact both histone modifications (Fig.3). Thus, the acetylation status of H4K16 may influence the extent of γ-H2AX formation. Upon TPX2 depletion, the decreased H4K16ac levels may prime the chromatin for excessive accumulation of γ-H2AX. H4K16 may also be part of a broader TPX2 and/or SIRT1-dependent chromatin remodeling program that affects phosphorylation of H2AX via unidentified mechanisms.

It remains unclear where (Fig.2F) and how TPX2 affects acetylation of H4K16. Based on our co-immunprecipitations of SIRT1 and TPX2 (Fig.3), we hypothesize that these two proteins may collaborate to regulate H4K16ac levels. In this context, it will be interesting to identify the specific genomic loci occupied by TPX2 and to determine whether these loci also contain SIRT1, H4K16ac, and/or ionizing radiation-inducible γ-H2AX. However, it remains unclear why TPX2 depletion does not impact other SIRT1 substrates, e.g. H3K9ac and H3K56ac [45], [61], [62]. Although TPX2/SIRT1 may specifically control loci that are enriched for H4K16ac but devoid of H3K9/56ac, TPX2 and SIRT1 could also act independently of each other. Further studies are required to define the mechanisms that regulate the TPX2-dependent H4K16ac/γ-H2AX levels.

Downstream of γ-H2AX, 53BP1 accumulates on chromatin flanking DNA lesions. This accumulation of 53BP1 also requires deacetylated H4K16, since acetylation of this histone-site inhibits binding of 53BP1's TUDOR domains to constitutively expressed di-methylated H4K20 [42], [47]. We found that depletion of TPX2 inhibits 53BP1 ionizing radiation-induced foci formation (Fig.4). Due to the increased γ-H2AX and constitutively decreased H4K16ac levels exhibited by TPX2-depleted cells, one might actually expect the opposite, i.e an increase in 53BP1 ionizing radiation-induced foci. However, our data are supported by several observations. First, mimicking globally deacetylated H4K16 does not increase 53BP1 foci formation either [47]. Second, excessive DNA damage caused by more than 2 Gy also interferes with formation of 53BP1 ionizing radiation-induced foci [57]. Such excessive damage that inhibits accumulation of 53BP1 at chromosomal breaks could be simulated by the pronounced DNA damage signaling of TPX2-depleted cells. Specifically, the increased γ-H2AX and decreased H4K16ac levels in TPX2-depleted cells (Figs.1-2) could signal more DNA damage than actually present and thus, misguide the 53BP1 system; (note that modulation of H4K16ac levels and γ-H2AX formation are both natural responses to chromosomal breakage; Fig.2A-B). This interpretation can be reconciled with the observation that a limited number of DNA lesions can still be efficiently decorated with 53BP1 upon depletion of TPX2 (Fig. 4C). In this context, one or two endogenous DNA double strand breaks with de-regulated H2AX phosphorylation and altered H4K16ac levels might not mimic beyond the threshold level of excessive DNA damage that exhausts the 53BP1 system [57].

Finally, since TPX2 has an active role during DNA damage response [15], we do not exclude that it contributes to the ionizing radiation-triggered modulation of H4K16ac levels [42], [47]. The idea that TPX2 may exert analogous chromatin modifying functions during physiological and DNA damaged contexts is exciting, novel, but not unprecedented. The ATM kinase that generates the majority of γ-H2AX during DNA damage response [16], [32] is also implicated in phosphorylation of H2AX on undamaged mitotic chromatin. The latter has been demonstrated to be important for chromosomal separation [63]. Furthermore, BRCA1-mediated ubiquitination of H2A, known to maintain integrity of heterochromatin [14], might also be important during DNA damage response. Downstream of γ-H2AX signaling, ubiquitination of DNA double strand break-flanking chromatin is essential for a functional DNA damage response [16], [17]. Interestingly, in BRCA1-depleted cells this ubiquitination of damaged chromatin is diminished [64]-[66]. TPX2 may be an addition to this list of factors that mediate analogous chromatin modifications during physiological conditions and DNA damage response.

## References

[1] Gruss OJ, Vernos I (2004) The mechanism of spindle assembly: functions of Ran and its target TPX2. J Cell Biol 166: 949–955. 15452138 10.1083/jcb.200312112 PMC2172015

[2] Gruss OJ, Wittmann M, Yokoyama H, Pepperkok R, Kufer T, et al (2002) Chromosome-induced microtubule assembly mediated by TPX2 is required for spindle formation in HeLa cells. Nat Cell Biol 4: 871–879. 12389033 10.1038/ncb870

[3] Wittmann T, Boleti H, Antony C, Karsenti E, Vernos I (1998) Localization of the kinesin-like protein Xklp2 to spindle poles requires a leucine zipper, a microtubule-associated protein, and dynein. J Cell Biol 143: 673–685. 9813089 10.1083/jcb.143.3.673 PMC2148133

[4] Wittmann T, Wilm M, Karsenti E, Vernos I (2000) TPX2, A novel Xenopus MAP involved in spindle pole organization. J Cell Biol 149: 1405–1418. 10871281 10.1083/jcb.149.7.1405 PMC2175143

[5] Bayliss R, Sardon T, Ebert J, Lindner D, Vernos I, et al (2004) Determinants for Aurora-A activation and Aurora-B discrimination by TPX2. Cell Cycle 3: 404–407. 14752279

[6] Bird AW, Hyman AA (2008) Building a spindle of the correct length in human cells requires the interaction between TPX2 and Aurora A. J Cell Biol. 182: 289–300. 10.1083/jcb.200802005 PMC2483532 18663142

[7] Kufer TA, Sillje HH, Korner R, Gruss OJ, Meraldi P, et al (2002) Human TPX2 is required for targeting Aurora-A kinase to the spindle. J Cell Biol 158: 617–623. 12177045 10.1083/jcb.200204155 PMC2174010

[8] Neumayer G, Belzil C, Gruss OJ, Nguyen MD (2014) TPX2: of spindle assembly, DNA damage response, and cancer. Cell Mol Life Sci 71: 3027–3047. 24556998 10.1007/s00018-014-1582-7 PMC11114040

[9] Goshima G (2011) Identification of a TPX2-like microtubule-associated protein in Drosophila. PLoS One 6: e28120. 22140519 10.1371/journal.pone.0028120 PMC3227607

[10] Kahana JA, Cleveland DW (2001) Cell cycle. Some importin news about spindle assembly. Science 291: 1718–1719. 11253198 10.1126/science.1059765

[11] Petrovska B, Jerabkova H, Kohoutova L, Cenklova V, Pochylova Z, et al (2013) Overexpressed TPX2 causes ectopic formation of microtubular arrays in the nuclei of acentrosomal plant cells. J Exp Bot 64: 4575–4587. 24006426 10.1093/jxb/ert271 PMC3808333

[12] Joukov V, Groen AC, Prokhorova T, Gerson R, White E, et al (2006) The BRCA1/BARD1 heterodimer modulates ran-dependent mitotic spindle assembly. Cell 127: 539–552. 17081976 10.1016/j.cell.2006.08.053

[13] Maxwell CA, Benitez J, Gomez-Baldo L, Osorio A, Bonifaci N, et al (2011) Interplay between BRCA1 and RHAMM regulates epithelial apicobasal polarization and may influence risk of breast cancer. PLoS Biol 9: e1001199. 22110403 10.1371/journal.pbio.1001199 PMC3217025

[14] Zhu Q, Pao GM, Huynh AM, Suh H, Tonnu N, et al (2011) BRCA1 tumour suppression occurs via heterochromatin-mediated silencing. Nature 477: 179–184. 21901007 10.1038/nature10371 PMC3240576

[15] Neumayer G, Helfricht A, Shim SY, Le HT, Lundin C, et al (2012) Targeting Protein for Xenopus kinesin like protein 2 (TPX2) regulates gamma-H2AX levels upon ionizing radiation. J Biol Chem 287: 42206–42222. 23045526 10.1074/jbc.M112.385674 PMC3516765

[16] van Attikum H, Gasser SM (2009) Crosstalk between histone modifications during the DNA damage response. Trends Cell Biol 19: 207–217. 19342239 10.1016/j.tcb.2009.03.001

[17] Stewart GS (2009) Solving the RIDDLE of 53BP1 recruitment to sites of damage. Cell Cycle 8: 1532–1538. 19372751 10.4161/cc.8.10.8351

[18] Huen MS, Chen J (2008) The DNA damage response pathways: at the crossroad of protein modifications. Cell Res 18: 8–16. 18087291 10.1038/cr.2007.109

[19] Polo SE, Jackson SP (2011) Dynamics of DNA damage response proteins at DNA breaks: a focus on protein modifications. Genes Dev 25: 409–433. 21363960 10.1101/gad.2021311 PMC3049283

[20] Lou Z, Minter-Dykhouse K, Franco S, Gostissa M, Rivera MA, et al (2006) MDC1 maintains genomic stability by participating in the amplification of ATM-dependent DNA damage signals. Mol Cell 21: 187–200. 16427009 10.1016/j.molcel.2005.11.025

[21] Hanahan D, Weinberg RA (2000) The hallmarks of cancer. Cell 100: 57–70. 10647931 10.1016/s0092-8674(00)81683-9

[22] Hanahan D, Weinberg RA (2011) Hallmarks of cancer: the next generation. Cell 144: 646–674. 21376230 10.1016/j.cell.2011.02.013

[23] Rogakou EP, Boon C, Redon C, Bonner WM (1999) Megabase chromatin domains involved in DNA double-strand breaks in vivo. J Cell Biol 146: 905–916. 10477747 10.1083/jcb.146.5.905 PMC2169482

[24] Rogakou EP, Pilch DR, Orr AH, Ivanova VS, Bonner WM (1998) DNA double-stranded breaks induce histone H2AX phosphorylation on serine 139. J Biol Chem 273: 5858–5868. 9488723 10.1074/jbc.273.10.5858

[25] Stiff T, O′Driscoll M, Rief N, Iwabuchi K, Lobrich M, et al (2004) ATM and DNA-PK function redundantly to phosphorylate H2AX after exposure to ionizing radiation. Cancer Res 64: 2390–2396. 15059890 10.1158/0008-5472.can-03-3207

[26] Nakada S, Chen GI, Gingras AC, Durocher D (2008) PP4 is a gamma H2AX phosphatase required for recovery from the DNA damage checkpoint. EMBO Rep 9: 1019–1026. 18758438 10.1038/embor.2008.162 PMC2527856

[27] Chowdhury D, Xu X, Zhong X, Ahmed F, Zhong J, et al (2008) A PP4-phosphatase complex dephosphorylates gamma-H2AX generated during DNA replication. Mol Cell 31: 33–46. 18614045 10.1016/j.molcel.2008.05.016 PMC3242369

[28] Macurek L, Lindqvist A, Voets O, Kool J, Vos HR, et al (2010) Wip1 phosphatase is associated with chromatin and dephosphorylates gammaH2AX to promote checkpoint inhibition. Oncogene 29: 2281–2291. 20101220 10.1038/onc.2009.501

[29] Douglas P, Zhong J, Ye R, Moorhead GB, Xu X, et al (2010) Protein phosphatase 6 interacts with the DNA-dependent protein kinase catalytic subunit and dephosphorylates gamma-H2AX. Mol Cell Biol 30: 1368–1381. 20065038 10.1128/MCB.00741-09 PMC2832507

[30] Moon SH, Lin L, Zhang X, Nguyen TA, Darlington Y, et al (2010) Wild-type p53-induced phosphatase 1 dephosphorylates histone variant gamma-H2AX and suppresses DNA double strand break repair. J Biol Chem 285: 12935–12947. 20118229 10.1074/jbc.M109.071696 PMC2857113

[31] Cha H, Lowe JM, Li H, Lee JS, Belova GI, et al (2010) Wip1 directly dephosphorylates gamma-H2AX and attenuates the DNA damage response. Cancer Res 70: 4112–4122. 20460517 10.1158/0008-5472.CAN-09-4244 PMC2904079

[32] Kurz EU, Lees-Miller SP (2004) DNA damage-induced activation of ATM and ATM-dependent signaling pathways. DNA Repair (Amst) 3: 889–900. 15279774 10.1016/j.dnarep.2004.03.029

[33] Cowell IG, Sunter NJ, Singh PB, Austin CA, Durkacz BW, et al (2007) gammaH2AX foci form preferentially in euchromatin after ionising-radiation. PLoS One 2: e1057. 17957241 10.1371/journal.pone.0001057 PMC2020439

[34] Kim JA, Kruhlak M, Dotiwala F, Nussenzweig A, Haber JE (2007) Heterochromatin is refractory to gamma-H2AX modification in yeast and mammals. J Cell Biol 178: 209–218. 17635934 10.1083/jcb.200612031 PMC2064441

[35] Allis CD, Jenuwein T, Reinberg D (2007) Epigenetics. Cold Spring Harbor, N.Y.: Cold Spring Harbor Laboratory Press.

[36] Wang RH, Sengupta K, Li C, Kim HS, Cao L, et al (2008) Impaired DNA damage response, genome instability, and tumorigenesis in SIRT1 mutant mice. Cancer Cell 14: 312–323. 18835033 10.1016/j.ccr.2008.09.001 PMC2643030

[37] Gupta A, Sharma GG, Young CS, Agarwal M, Smith ER, et al (2005) Involvement of human MOF in ATM function. Mol Cell Biol 25: 5292–5305. 15923642 10.1128/MCB.25.12.5292-5305.2005 PMC1140595

[38] Ikura T, Ogryzko VV, Grigoriev M, Groisman R, Wang J, et al (2000) Involvement of the TIP60 histone acetylase complex in DNA repair and apoptosis. Cell 102: 463–473. 10966108 10.1016/s0092-8674(00)00051-9

[39] Yuan Z, Zhang X, Sengupta N, Lane WS, Seto E (2007) SIRT1 regulates the function of the Nijmegen breakage syndrome protein. Mol Cell 27: 149–162. 17612497 10.1016/j.molcel.2007.05.029 PMC2679807

[40] Peng L, Ling H, Yuan Z, Fang B, Bloom G, et al (2012) SIRT1 negatively regulates the activities, functions, and protein levels of hMOF and TIP60. Mol Cell Biol 32: 2823–2836. 22586264 10.1128/MCB.00496-12 PMC3416197

[41] Yamagoe S, Kanno T, Kanno Y, Sasaki S, Siegel RM, et al (2003) Interaction of histone acetylases and deacetylases in vivo. Mol Cell Biol 23: 1025–1033. 12529406 10.1128/MCB.23.3.1025-1033.2003 PMC140702

[42] Tang J, Cho NW, Cui G, Manion EM, Shanbhag NM, et al (2013) Acetylation limits 53BP1 association with damaged chromatin to promote homologous recombination. Nat Struct Mol Biol 20: 317–325. 23377543 10.1038/nsmb.2499 PMC3594358

[43] Sharma GG, So S, Gupta A, Kumar R, Cayrou C, et al (2010) MOF and histone H4 acetylation at lysine 16 are critical for DNA damage response and double-strand break repair. Mol Cell Biol 30: 3582–3595. 20479123 10.1128/MCB.01476-09 PMC2897562

[44] Wu J, Chen Y, Lu LY, Wu Y, Paulsen MT, et al (2011) Chfr and RNF8 synergistically regulate ATM activation. Nat Struct Mol Biol 18: 761–768. 21706008 10.1038/nsmb.2078 PMC3130800

[45] Vaquero A, Scher M, Lee D, Erdjument-Bromage H, Tempst P, et al (2004) Human SirT1 interacts with histone H1 and promotes formation of facultative heterochromatin. Mol Cell 16: 93–105. 15469825 10.1016/j.molcel.2004.08.031

[46] Vaquero A, Sternglanz R, Reinberg D (2007) NAD+-dependent deacetylation of H4 lysine 16 by class III HDACs. Oncogene 26: 5505–5520. 17694090 10.1038/sj.onc.1210617

[47] Hsiao KY, Mizzen CA (2013) Histone H4 deacetylation facilitates 53BP1 DNA damage signaling and double-strand break repair. J Mol Cell Biol 5: 157–165. 23329852 10.1093/jmcb/mjs066

[48] Berger SM, Pesold B, Reber S, Schonig K, Berger AJ, et al (2010) Quantitative analysis of conditional gene inactivation using rationally designed, tetracycline-controlled miRNAs. Nucleic Acids Res 38: e168. 20639530 10.1093/nar/gkq616 PMC2943624

[49] Ota H, Tokunaga E, Chang K, Hikasa M, Iijima K, et al (2006) Sirt1 inhibitor, Sirtinol, induces senescence-like growth arrest with attenuated Ras-MAPK signaling in human cancer cells. Oncogene 25: 176–185. 16170353 10.1038/sj.onc.1209049

[50] Tjeertes JV, Miller KM, Jackson SP (2009) Screen for DNA-damage-responsive histone modifications identifies H3K9Ac and H3K56Ac in human cells. EMBO J 28: 1878–1889. 19407812 10.1038/emboj.2009.119 PMC2684025

[51] Kagawa S, Gu J, Honda T, McDonnell TJ, Swisher SG, et al (2001) Deficiency of caspase-3 in MCF7 cells blocks Bax-mediated nuclear fragmentation but not cell death. Clin Cancer Res 7: 1474–1480. 11350920

[52] Janicke RU, Sprengart ML, Wati MR, Porter AG (1998) Caspase-3 is required for DNA fragmentation and morphological changes associated with apoptosis. J Biol Chem 273: 9357–9360. 9545256 10.1074/jbc.273.16.9357

[53] Rogakou EP, Nieves-Neira W, Boon C, Pommier Y, Bonner WM (2000) Initiation of DNA fragmentation during apoptosis induces phosphorylation of H2AX histone at serine 139. J Biol Chem 275: 9390–9395. 10734083 10.1074/jbc.275.13.9390

[54] Stewart GS, Panier S, Townsend K, Al-Hakim AK, Kolas NK, et al (2009) The RIDDLE syndrome protein mediates a ubiquitin-dependent signaling cascade at sites of DNA damage. Cell 136: 420–434. 19203578 10.1016/j.cell.2008.12.042

[55] van Attikum H, Gasser SM (2005) The histone code at DNA breaks: a guide to repair? Nat Rev Mol Cell Biol 6: 757–765. 16167054 10.1038/nrm1737

[56] Lukas C, Savic V, Bekker-Jensen S, Doil C, Neumann B, et al (2011) 53BP1 nuclear bodies form around DNA lesions generated by mitotic transmission of chromosomes under replication stress. Nat Cell Biol 13: 243–253. 21317883 10.1038/ncb2201

[57] Gudjonsson T, Altmeyer M, Savic V, Toledo L, Dinant C, et al (2012) TRIP12 and UBR5 suppress spreading of chromatin ubiquitylation at damaged chromosomes. Cell 150: 697–709. 22884692 10.1016/j.cell.2012.06.039

[58] Harrigan JA, Belotserkovskaya R, Coates J, Dimitrova DS, Polo SE, et al (2011) Replication stress induces 53BP1-containing OPT domains in G1 cells. J Cell Biol 193: 97–108. 21444690 10.1083/jcb.201011083 PMC3082192

[59] Giunta S, Belotserkovskaya R, Jackson SP (2010) DNA damage signaling in response to double-strand breaks during mitosis. J Cell Biol 190: 197–207. 20660628 10.1083/jcb.200911156 PMC2930281

[60] Giunta S, Jackson SP (2011) Give me a break, but not in mitosis: the mitotic DNA damage response marks DNA double-strand breaks with early signaling events. Cell Cycle 10: 1215–1221. 21412056 10.4161/cc.10.8.15334 PMC3117133

[61] Das C, Lucia MS, Hansen KC, Tyler JK (2009) CBP/p300-mediated acetylation of histone H3 on lysine 56. Nature 459: 113–117. 19270680 10.1038/nature07861 PMC2756583

[62] Li Z, Chen L, Kabra N, Wang C, Fang J, et al (2009) Inhibition of SUV39H1 methyltransferase activity by DBC1. J Biol Chem 284: 10361–10366. 19218236 10.1074/jbc.M900956200 PMC2667723

[63] McManus KJ, Hendzel MJ (2005) ATM-dependent DNA damage-independent mitotic phosphorylation of H2AX in normally growing mammalian cells. Mol Biol Cell 16: 5013–5025. 16030261 10.1091/mbc.E05-01-0065 PMC1237100

[64] Morris JR, Solomon E (2004) BRCA1: BARD1 induces the formation of conjugated ubiquitin structures, dependent on K6 of ubiquitin, in cells during DNA replication and repair. Hum Mol Genet 13: 807–817. 14976165 10.1093/hmg/ddh095

[65] Polanowska J, Martin JS, Garcia-Muse T, Petalcorin MI, Boulton SJ (2006) A conserved pathway to activate BRCA1-dependent ubiquitylation at DNA damage sites. EMBO J 25: 2178–2188. 16628214 10.1038/sj.emboj.7601102 PMC1462971

[66] Zhao GY, Sonoda E, Barber LJ, Oka H, Murakawa Y, et al (2007) A critical role for the ubiquitin-conjugating enzyme Ubc13 in initiating homologous recombination. Mol Cell 25: 663–675. 17349954 10.1016/j.molcel.2007.01.029

